# Overexpression of brachyury contributes to tumor metastasis by inducing epithelial-mesenchymal transition in hepatocellular carcinoma

**DOI:** 10.1186/s13046-014-0105-6

**Published:** 2014-12-14

**Authors:** Rui Du, Shanshan Wu, Xiaoning Lv, Henghu Fang, Sudong Wu, Jingbo Kang

**Affiliations:** Department of Radiation Oncology and Integrative Oncology, Navy General Hospital, 6# Fu Cheng Road, Beijing, 100037 People’s Republic of China; Department of Aviation and Diving, Navy General Hospital, Beijing, People’s Republic of China

**Keywords:** Hepatocellular carcinoma, Brachyury, Cancer metastasis, Epithelial-Mesenchymal Transition, PTEN/Akt/Snail-Dependent Pathway

## Abstract

**Aims:**

Brachyury overexpression has been reported in various human malignant neoplasms, but its expression and function in hepatocellular carcinoma progression and metastasis remains unknown. The present study aimed to evaluate the critical role of Brachyury in HCC metastasis.

**Methods:**

The expression of Brachyury in human HCC (SMMC7721, HepG2, FHCC98, and Hep3B) and control cell lines was analyzed using quantitative reverse-transcriptase polymerase chain reaction and immunoflourence methods. Cancerous tissues collected from patients with HCC (n = 112) were analyzed using immunohistochemical method; a microarray analysis of HCC tissues was performed to explore the clinicopathological variables of HCC. The migratory and invasive capacities of Brachyury-SMMC7721 and Brachyury-HepG2 transfected cells were evaluated using in vitro scratch wound healing and Matrigel invasion assays, respectively. Further, six-week-old male BALB/c nude mice (n = 10) model was used in vivo assay.

**Results:**

Elevated expression of Brachyury was detected in HCCs (62.5%) compared with that in adjacent nontumorous tissues. Clinicopathological analysis revealed a close correlation of Brachyury expression with distant metastasis and poor prognosis of HCC. Overexpression of Brachyury promoted epithelial-mesenchymal transition (EMT) and metastasis of HCC cells in vitro and in vivo. Brachyury overexpression enhanced Akt activation by inhibiting phosphatase and tensin homolog (PTEN), which led to subsequent stabilization of Snail, a critical EMT mediator.

**Conclusion:**

The study findings suggest that elevated Brachyury facilitates HCC metastasis by promoting EMT via PTEN/Akt/Snail-dependent pathway. Brachyury plays a pivotal role in HCC metastasis and may serve as a novel prognostic biomarker and therapeutic target.

## Introduction

Hepatocellular carcinoma (HCC) is ranked as the fifth most frequent cancer and third most frequent cause of cancer deaths in the world [1]. In spite of improvements in surveillance and clinical therapeutic strategy, long-term disease-free survival (DFS) of patients with HCC remains unsatisfactory due to tumor recurrence and metastasis of the primary tumor [2,3]. Metastasis is not only a complex process but also the major cause of cancer-related deaths [4]. Epithelial-mesenchymal transition (EMT) describes a series of events during which cells lose epithelial characteristics such as cell-layer organization and apical-basolateral polarization and acquire properties of mesenchymal or fibroblastoid cells including motility [5]. EMT is known to be a central mechanism responsible for invasiveness and metastasis of various cancers [6,7]. Increasing evidences suggest that activation of Rho-family of small GTPases, cytoskeletal rearrangement, and nuclear translocation of several transcription factors such as Snail and Twist play important roles in the processes of cancer cell metastasis through modulation of cancer cells EMT [8-10]. However, the underlying mechanisms of cancer-related EMT are not fully elucidated.

The T-box transcription factor, Brachyury, is vital for the formation and differentiation of posterior mesoderm and axial development in vertebrates [11]. Interestingly, Brachyury is located at 6q27, which is a frequently amplified region in HCC [12]. The function of Brachyury has extensively been characterized in mice and zebrafish [13,14]. An earlier study on Brachyury-mutant mice model without T protein reported in utero death with abnormal notochord, absent somites, and reduced allantois [15]. In zebrafish, the no-tail mutation (Ntl) is the homolog of Brachyury; the Ntl embryos die soon after hatching with lack notochords and tails, and they possess abnormal trunk somites. The T gene encodes a transcription factor that binds to a specific DNA element via its N-terminal region [14]. In humans, T-box transcription factors are major players in the regulation of the progenitors and their differentiated descendants [11,16]. Brachyury is expressed in the progenitor population throughout somitogenesis, suggesting that it plays an essential role in keeping the progenitor population viable [17]. Meanwhile, Brachyury is also dysregulated in various human malignant neoplasms [18,19]. Recently, Brachyury was demonstrated to induce EMT in human epithelial cells through induction Snail, Slug, and downstream signal [20]. An earlier study on human lung carcinoma cells (in vitro and in vivo) has demonstrated that overexpressed Brachyury divides at slower rates than those with low-expressed Brachyury, a phenomenon associated with marked downregulation of cyclin D1, phosphorylated Rb, and CDKN1A (p21) [21]. Another study on oral squamous cell carcinoma cells demonstrated that the expression of Brachyury was correlated with EMT and was significantly associated with lymph node and distant metastasis [22]. The above evidences indicate that Brachyury may be a critical regulator of carcinogenesis and tumor metastases in different cancers. Until now, no studies have reported its roles in HCC. Hence, the present study aimed to evaluate the Brachyury expression in patients with HCC and in HCC cell lines and its clinicopathological significance in HCC.

## Materials and methods

### Cell culture

Human simian virus 40-transformed immortal liver epithelial cells (QZG) and HCC cell lines SMMC7721, HepG2, FHCC98, and Hep3B were purchased (Bei Na Biotechnology Research Institute, Beijing, China) and preserved [23]. Cells were maintained at 37°C with 5% carbon dioxide in Dulbecco’s Modified Eagle’s Medium (DMEM) (Gibco® Life Technologies, Grand Island, USA), supplemented with 50 units/ml penicillin, 50 mg/ml streptomycin, and 10% fetal calf serum (Gibco® Life Technologies, Burlington, Canada).

### Patients and tissue samples

Patients (n = 40; aged between 32 and 79 years with a mean age of 50.12 ± 15.02 years), who underwent hepatectomy for HCC between January 2006 and August 2008 in the Department of General Surgery, Xijing Hospital (Xi’an, China), were included in the Brachyury and vimentin correlation analysis. Tissue chips for HCC primary tumors were purchased from National Engineering Center for Biochip Design, Shanghai Biochip Co. Ltd., Shanghai, China. The tissue chips (n = 72) were used in the clinicopathological correlation and survival analysis. None of these patients received preoperative chemotherapy or radiotherapy. All tumor samples were derived from dissected tumor tissues and were composed of more than 90% of tumor cells without necrosis. The surgical specimens (both tumor and adjacent nontumorous tissues) were processed immediately after the operation and snap-frozen in liquid nitrogen for protein, DNA, and ribonucleic acid (RNA) extraction. All patients with HCC gave written informed consent on the use of clinical specimens for medical research. Studies using human tissues were reviewed and approved by the Committees for Ethical Review of research involving human subjects of Fourth Military Medical University, Xi’an, China.

### Immunohistochemistry (IHC) and Immunofluorescence (IF)

For IF analysis, SMMC7721 and HepG2 cells were cultured on sterile glass coverslips in 24-well plates. The slides were fixed with 95% alcohol for 15 minutes at room temperature. The coverslips were washed with phosphate-buffered saline (PBS) and permeabilized for 5 minutes with 0.5% Triton X-100 in PBS. The cells were then incubated with primary antibodies including anti-E-cadherin and anti-vimentin (1:100 ratios; Santa Cruz Biotechnology, Santa Cruz, USA) after blocking with 10% normal goat or rabbit serum for 1 h. The slides were incubated with fluorescein isothiocyanate-conjugated goat anti-mouse or anti-rabbit Immunoglobulin G (IgG) as the secondary antibody at room temperature for 1 h. Slides were examined with a Nikon (Melville, USA) Eclipse TE300 fluorescence microscope, and the pictures were taken with a SPOT Diagnostic CCD camera (SPOT™ Imaging Solutions, Sterling Heights, USA).

The paraffin-embedded tissue blocks were sectioned for IHC analysis. In brief, tissue sections were deparaffinized and rehydrated. The endogenous peroxidase activity was blocked with 3% hydrogen peroxide for 10 minutes. For antigen retrieval, slides were immersed in 10 mM citrate buffer (pH 6.0) and boiled for 15 minutes in a microwave oven. Nonspecific binding was blocked by 5% normal goat serum for 10 minutes. The slides were incubated with anti-Brachyury polyclonal antibody (diluted at 1:500 ratio; Abcam, USA), anti-E-cadherin antibody, and anti-Snail polyclonal antibody (diluted at 1:200 ratio; Santa Cruz Biotechnology, Santa Cruz, USA) at 4°C overnight in a moist chamber. The slides were sequentially incubated with biotinylated goat anti-mouse IgG (1:100 dilution; Santa Cruz Biotechnology, Santa Cruz, USA) and then with streptavidin-peroxidase conjugate, each for 30 minutes at room temperature. Isotope-matched human IgG was used in each case as a negative control. Finally, the 3, 5-diaminobenzidine Substrate Kit (Dako, Glostrup, Denmark) was used for color development followed by Mayer’s hematoxylin counter staining. The slides were counterstained with hematoxylin, dehydrated and mounted. Tumor cell proportions were scored as follows: 0 (no positive tumor cells); 1 (<25% positive tumor cells); 2 (25–50% positive tumor cells); and 3 (>50% positive tumor cells). Staining intensity was graded according to the following standard: 0 (no staining); 1 (weak staining = light yellow); 2 (moderate staining = yellow brown) and 3 (strong staining = brown). The staining index (SI) was calculated as the product of the staining intensity score and the proportion of positive tumor cells. Using this method, we evaluated nuclear Brachyury expression in HCC tumor tissues and adjacent nontumorous ones by determining the SI, with scores of 0, 1, 2, 3, 4, 6, 8, 9. Only 2+ and 3+ was considered as a positive IHC result [20].

### Quantitative real-time polymerase chain reaction (qRT-PCR)

Total RNA was isolated using Trizol reagent (Life Technologies, China), reverse transcribed with TaKaRa reverse transcription Kit (TaKaRa, Dalian, China) starting with 2 μg total RNA from each sample per manufacturer’s instructions. The messenger ribonucleic acid (mRNA) expressions of Brachyury and housekeeping gene glyceraldehyde-3-phosphate dehydrogenase (GAPDH) were analyzed by quantitative RT-PCR using a real-time LightCycler® rapid thermal cycler (Roche Molecular Biochemicals LightCycler System, Shanghai, China). Specific Brachyury primers 5’-ACTGAGAATCAGCCGGACTT-3’ (forward) 5’-CTGCACTGCAAAGAACCACT-3’ (reverse) and internal control GAPDH primers 5’-GCACCGTCAAGGCTGAGAAC-3’ (forward) and 5’-TGGTGAAGACGCCAGTGGAT-3’ (reverse) were used for mRNA amplifications. For quantitative RT-PCR, 1 μl of gene primers with SYBR® Green (Applied Biosystems, China) in 25 μl of reaction volume was used. The MTBP mRNA levels were normalized to GAPDH mRNA according to the following formula: 2^-(CT target – CT GAPDH)^, where CT was the threshold cycle.

### Plasmids and cell transfection

Human Brachyury was amplified by the PCR using cDNA from SMMC7721, confirmed by sequencing and subcloned into pCDNA3.1 expression vector(Invitrogen, Carlsbad, USA). Cell transfection was carried out by plating 2 × 10^5^ HepG2 and SMMC7721 cells on six-well plate and transfected with 800-ng constructs using Lipofectamine™ 2000 (Invitrogen Life Technology, Carlsbad, USA) per manufacturer’s instructions. Briefly, cells grew 70% to 90% confluence before transfection. The pcDNA3.1-Brachyury and pcDNA3.1-control were transfected using Lipofectamine™ 2000 in DMEM. The cells were selected for more than 4 weeks by incubation with G418 (Invitrogen, 400 ng/ml for SMMC-7721 and 600 ng/ml for HepG2) for overexpression clones. Stable single clones were selected and Brachyury expression assessed using western blotting. Transient transfection of pcDNA3.1-Brachyury and the control constructs into HCC cells were performed using Lipofectamine 2000.

### Protein preparation and Western blot analysis

Harvested cells (2 × 10^6^) were placed in 1.5-ml Eppendorf tubes and homogenized with 400 μl lysis buffer (50 mM Tris-hydrochloride, pH 8.0, 150 mM sodium chloride, 0.1% sodium dodecyl sulfate, 1% Nonidet P-40, 0.5% sodium deoxycholate, 0.02% sodium azide, 100 μg/ml phenylmethanesulfonyl fluoride, and 1 μg/ml aprotinin). Cell lysates were centrifuged at 4°C for 5 minutes at 10,000 rpm, and the protein-containing supernatant was placed in fresh tubes and quantified using the Bradford protein assay.

Western blot analysis was performed as described previously [24]. In brief, the membranes with total protein were incubated with primary antibodies: anti-Brachyury and anti-Snail (1:1000 dilutions; Cell Signaling Technology, Danvers, USA), anti-E-cadherin, anti-γ-catenin, anti-vimentin, anti-Akt, anti-p-Akt, and anti-PTEN (1:200 dilution; Santa Cruz Biotechnology, Santa Cruz, USA). After repeated washing, the membranes were incubated with horseradish-peroxidase-conjugated anti-rabbit or anti-mouse secondary antibody (1:2000 dilution; Santa Cruz Biotechnology, Santa Cruz, USA). The bands were visualized using the enhanced chemiluminescence detection system (Amersham Pharmacia Biotech, Piscataway, NJ, USA) and exposed to Kodak X-OMAT film (Rochester, USA). β-actin was used as an internal sample loading control. Autoradiograms were quantified using densitometry (Bio Image Intelligent Quantifier software, Bio Image Systems Inc., Jackson, MI, USA). Relative protein levels were calculated by normalization to the amount of β-actin protein.

### Wound healing and invasion assays

Wound-healing assay and invasion assay were performed as described previously [12]. Cell migration was assessed by measuring the movement of cells into a scraped; a cellular area was created using a 200-μl pipette tube, and the spread of wound closure was observed after 24 hours and photographed under a microscope. The % wound healing was quantified by the space of migration tumor cells at 24 h after scrambled /the space of wound at 0 h × 100%. Cell invasion was quantified in vitro using Transwell chambers with polycarbonate membrane filters (8-μm pore size) coated with a Matrigel™ (Sigma, St. Louis, USA). Briefly, 48 hours after transfection, cells were washed twice using DMEM and seeded in triplicate in the inner chamber of the insert containing 200 μl of serum-free medium. About 700 μl of medium containing 10% fetal bovine serum was added to the lower chamber. The plates were incubated for 24 hours at 37°C. Then, the noninvading cells from the interior of the inserts were gently removed using a cotton-tipped swab. The cells that had invaded into the bottom surface of the filter were fixed with methanol and stained with hematoxylin. The invasive ability was determined by counting the penetrating cells under a microscope at × 200 magnification on 10 random fields in each well. The experiments were performed in triplicate.

### In vivo metastasis assay

Six-week-old male BALB/c nude mice (n = 10) were randomized into two groups. HepG2/Brachyury or control cells (2 × 10^6^) were injected into the tail vein of nude mice. Four mice were sacrificed 6 weeks post inoculation and consecutive sections of the whole lung were subjected to hematoxylin and eosin staining. All of the metastatic foci in lung were calculated microscopically to evaluate the development of pulmonary metastasis. The remaining mice were monitored for survival analysis.

### Statistical analysis

Unless otherwise indicated, data were presented as mean ± standard deviation of three independent experiments. SPSS statistical package for Windows (Version 12.0, SPSS Inc. Chicago, IL, USA) was used for data analysis. Based on staining intensities, the Brachyury immunoreactivity was scored as negative (0 to 1) and positive (2 to 3) according to a previously reported semiquantitative scoring method [25]. The clinicopathological features of Brachyury-positive and Brachyury-negative patients were compared using Pearson χ^2^ test for categorical variables and independent Student’s t-test for continuous data. Comparisons of three or more variables were performed using one-way analysis of variance followed by Fisher’s protected least significant difference test. Kaplan-Meier plots and log-rank tests were used for survival analysis. The DFS times were calculated from data of curative surgery to HCC recurrence, death, or the last follow-up data. For tissue microarray analysis, based on IHC scores, Brachyury protein levels in primary HCC tissues and their matched metastatic tissues were compared using Wilcoxon signed-rank test. The correlation between mRNA levels of Brachyury and Snail was analyzed using Pearson χ^2^ test. A P value less than 0.05 was considered statistically significant.

## Results

### Expression of brachyury is upregulated in human HCCs

**Figure 1:**
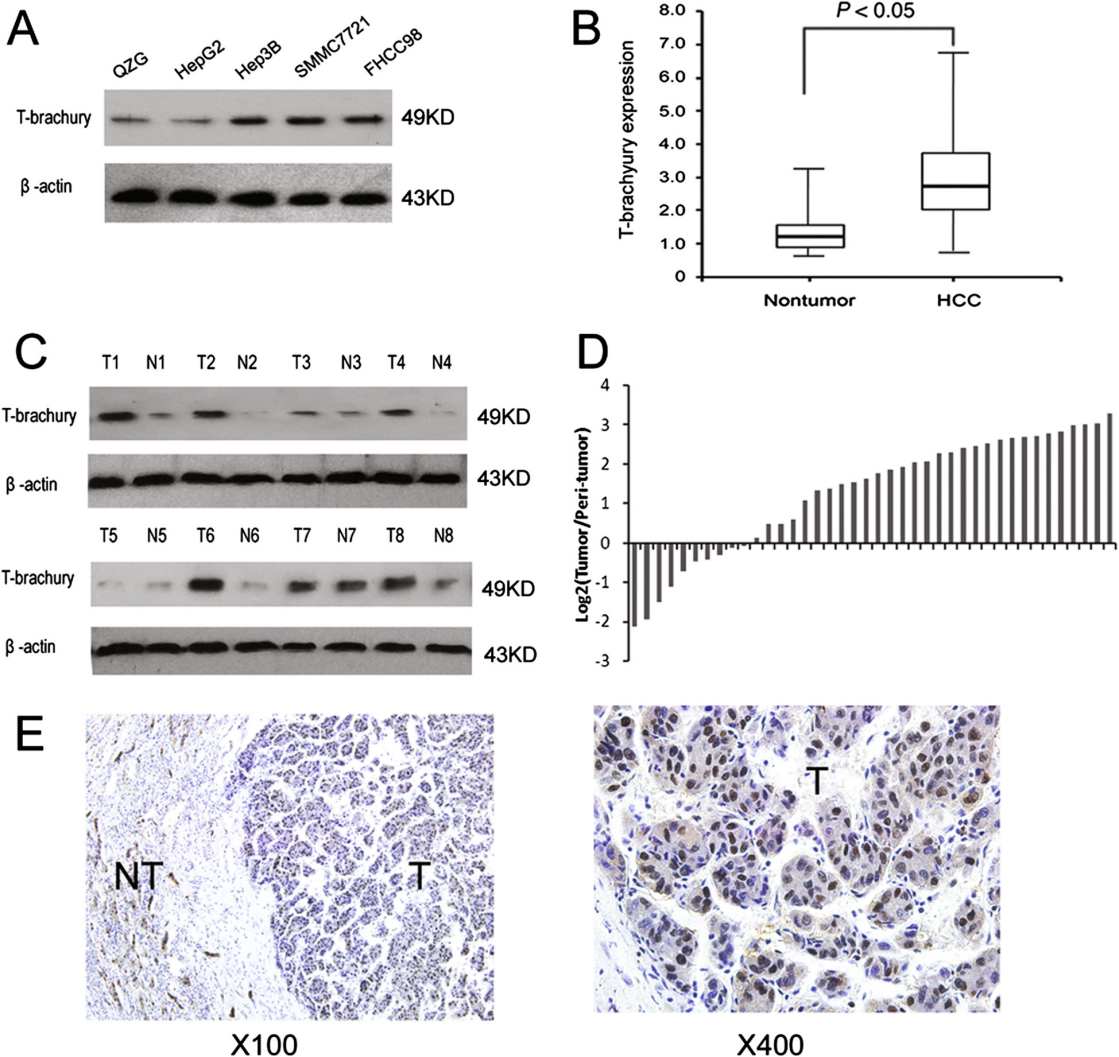
**Expression of Brachyury is up-regulated in human HCCs. (A)** Expression of Brachyury in one human normal liver cell lines (QZG) and four hepatoma cell lines was examined by western blot assay. **(B)** Brachyury expression in 40 pairs of HCC and the surrounding tissues were detected by real-time PCR. **(C)** Representative western blot showing the expression of Brachyury protein in tumor tissue (T) and paired peri-tumor tissue (N) from eight HCC patients. **(D)** Relative immunohistochemical staining of Brachyury expression in paired HCC tissue samples (n = 112), and the representative view **(E)**.

To explore the role of Brachyury in HCC development, the expression of Brachyury in various human HCC cell lines were evaluated. As shown in Figure 1A, elevated expression of Brachyury was observed in all four HCC cell lines compared with that in QZG cell lines. Brachyury mRNA was significantly increased in HCCs relative to paired noncancerous tissues of 40 patients (Figure 1B), which was further confirmed by Western blot assay (Figure 1C,D). Immunohistochemical analysis showed little positive staining of Brachyury in nontumorous liver cells, but strong nuclei staining of Brachyury (upregulated) in 62.5% (70/112) of the patients with HCC. Brachyury was predominantly located in the nuclei of hepatocytes and tumor cells (Figure 1E). Brachyury positive expression was found in tumor tissues at a value (62.5%, 70 of 112 patients) higher than that in adjacent cirrhosis tissues (23.2%, 26 of 112 patients) (P < 0.05).

### Brachyury overexpression predicts poor prognosis of HCC

**Figure 2:**
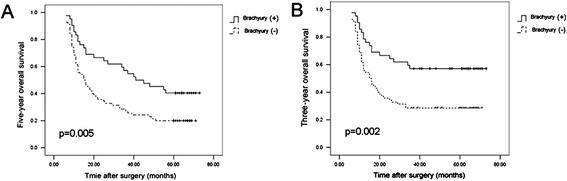
**Brachyury overexpression predicts poor prognosis of HCC. (A)** HCC patients were divided into Brachyury-positive expression group (n = 70) and Brachyury-negative expression group (n = 40). The 5-year overall of 112 HCC patients were compared between the low and high Brachyury groups. **(B)** The 3-year overall of 112 HCC patients were compared between the low and high Brachyury groups.

To investigate the clinical significance of Brachyury overexpression in HCC, microarray analysis of HCC tissues from 72 patients who underwent liver resection and 40 PPEP tissues was performed (Table 1). The Mann-Whitney U test was used to assess the correlations between the staining intensity of Brachyury protein and clinicopathologic variables of HCC. As shown in Table 2, the Brachyury expression in patients with HCC was correlated with tumor size (P = 0.037), intrahepatic invasion (P = 0.025), distance metastasis (P = 0.039). However, the Brachyury protein staining intensity showed no significant relationship with gender, age, α-fetoprotein and HBV infection. Based on the results from IHC, all 112 patients with HCC were divided into two groups: Brachyury-positive expression group (n = 70) and Brachyury-negative expression group (n = 42). Patients in positive expression group exhibited shorter 5-year overall survival (OS, median OS were 16 and 43 months, respectively, difference of 27 months, P = 0.004) than those in negative expression group (Figure 2A). Consistently, the 3-year OS rate after surgery was much lower in Brachyury-positive group than that in Brachyury-negative group (Figure 2B). Thus, Brachyury overexpression could serve as a valuable predicting factor for recurrence and poor survival of patients with HCC.

**Table 1:** **Summary of clinicopathologic variables**

**Characteristic**	**No. of patients**
Patients	112
Gender	
Male	92
Female	20
Age	
< 55	72
≥ 55	40
AFP (μg/L)	
< 100	47
≥ 100	65
HBV	
Positive	91
Negative	21
Liver Cirrhosis	
Yes	66
No	46
Tumor size (cm)	
< 3	37
≥ 3	75
Intrahepatic metastasis	
Y	41
N	71
Distant metastasis	
Y	27
N	85
TNM stage	
I	27
II	62
III	23

**Table 2:** **Relationship between Brachyury expression and clinicopathologic features of HCC patients**

**Features**		**Relative Brachyury expression**		**p Value**
		**High**	**Low**	
Sex	Male	60	32	0.797
	Female	12	8	
Age	<55	45	27	0.683
	≥55	27	13	
AFP	<100	32	15	0.551
	≥100	40	25	
HBV	Positive	58	32	0.807
	Negative	13	8	
Liver Cirrhosis	Yes	47	19	0.075
	No	25	21	
Tumor size (cm)	<3	19	18	**0.037**
	≥3	53	22	
Intrahepatic Metastasis	Y	32	9	**0.025**
	N	40	31	
Distant Metastasis	Y	22	5	**0.039**
	N	50	35	
TNM Stage	I	15	12	0.401
	II	40	22	
	III	17	6

### Brachyury enhances invasive and metastatic potential of HCC

**Figure 3:**
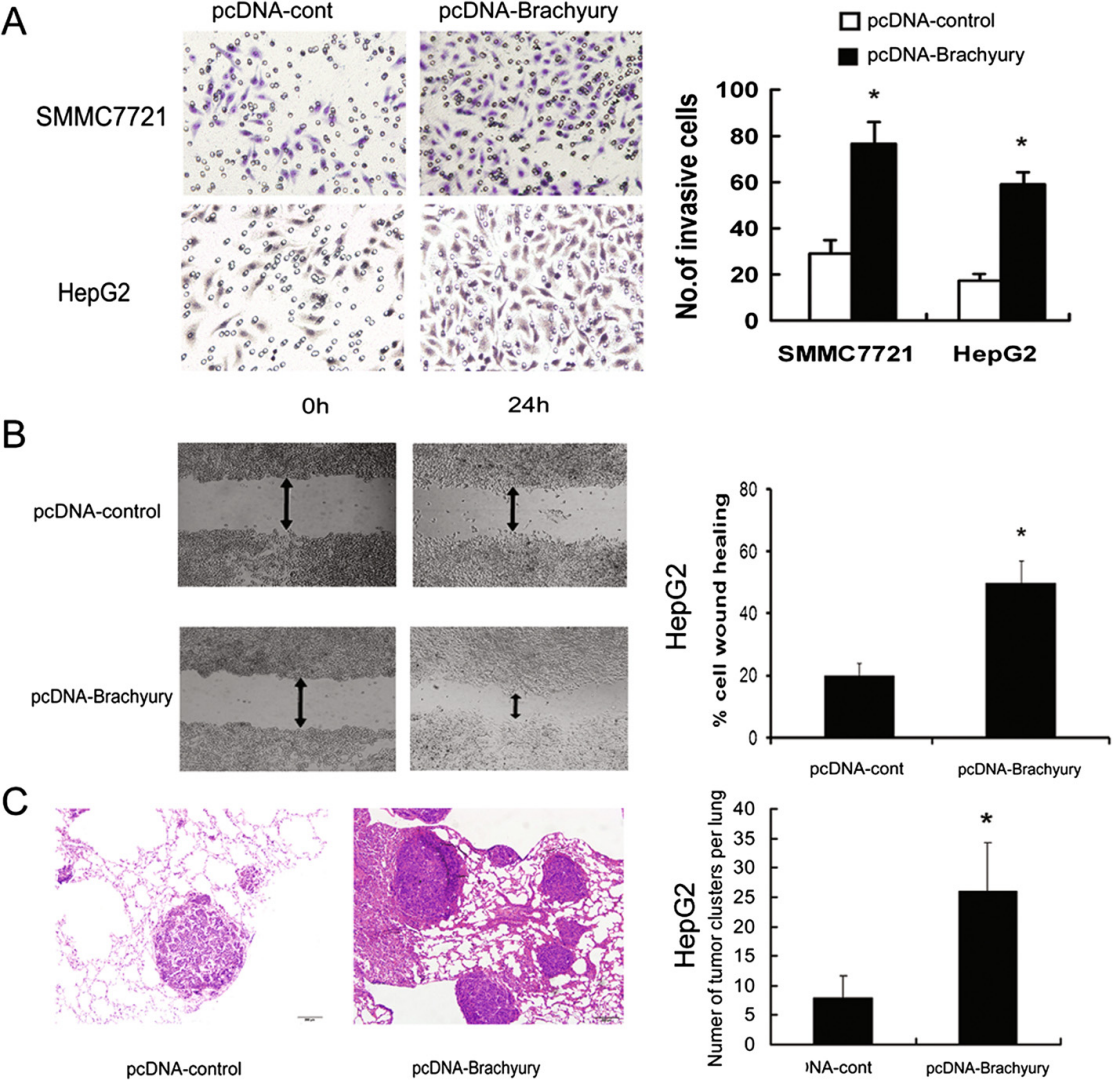
**Brachyury enhances metastatic potential of HCC cells. (A)** The invasive properties of the cells were analyzed in by an invasion assay using a Matrigelcoated Boyden chamber. Migrated cells were plotted as the average number of cells per field of view from 3 different experiments, as described in Materials and Methods. **(B)** The cell migration rate between SMMC-7721/Brachyury and SMMC-7721/GFP cells was compared by wound-healing assay. Microscopic observation was recorded at 0 and 24 hours after scratching the cell layer. **(C)** Representative view of lung tissue sections from each group are shown (H&E stain; magnification × 100). The number of lung metastatic foci in each group (n = 10) of HepG2/Brachyury or HepG2/GFP xenografted mice was calculated microscopically 6 weeks after tail vein injection. *p < 0.05.

The EMT promotes tumor progression by increasing the invasiveness and migratory capacity of the tumor cells. To test whether Brachyury-SMMC7721 and Brachyury-HepG2 cells acquired greater migratory and invasive capabilities, in vitro scratch wound healing and Matrigel invasion assays were performed. The Matrigel invasion assay showed that the invasiveness of the Brachyury-expressing cells was significantly higher than empty vector-transfected cells (P < 0.001, independent Student’s t test; Figure 3A). Similarly, the wound healing assay demonstrated that the ectopic expression of Brachyury increased the cell motility in both Brachyury-SMMC7721 and Brachyury-HepG2 cells compared with Vec-SMMC7721 and Vec-HepG2 cells (Figure 3B). To further verify the effect of Brachyury on tumor metastasis, in vivo metastasis assay was performed in nude mice. Brachyury-HepG2 cells were injected into nude mice via tail vein, Empty vector trasfected cells were used as controls. Six weeks later, more and larger micrometastatic lesions were microscopically detected in the lungs of nude mice inoculated with Brachyury-HepG2 compared to that in the control group (P < 0.001, independent Student’s t test; Figure 3C). These data suggested that Brachyury promoted HCC invasion and metastasis.

### Brachyury promotes EMT in HCC cells

**Figure 4:**
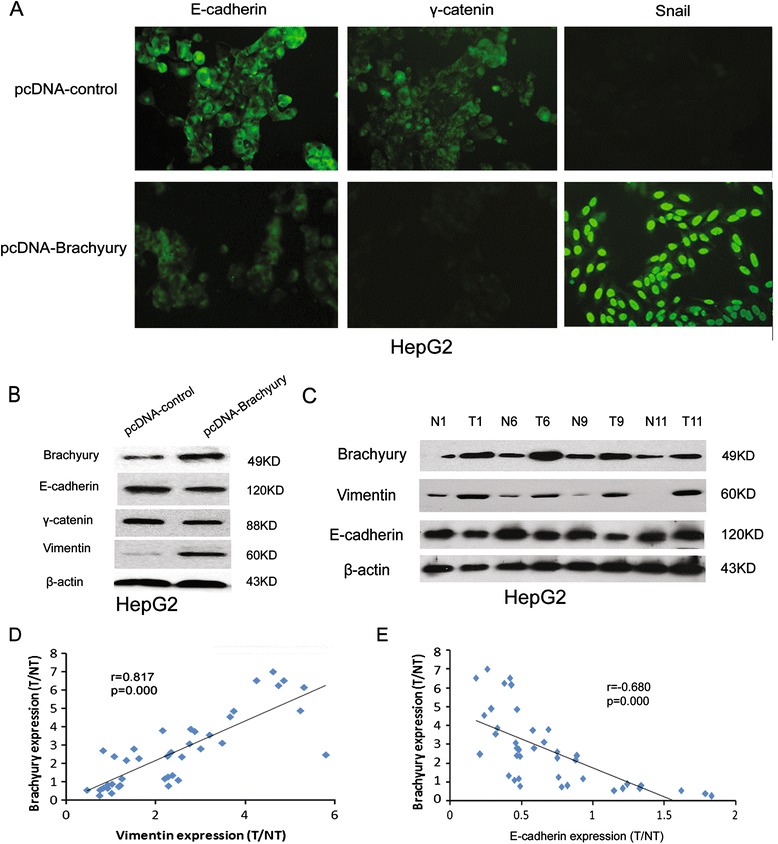
**Brachyury promotes EMT in HCC cells. (A)** HepG2/ Brachyury and control cells were cultured on Poly-L-Lysine coated glass coverslips for 24 hours followed by immunofluorescence assay. **(B)** Relative expression of E-cadherin, γ-catenin, and Vimentin in HepG2/ Brachyury cells compared to control cells. **(C)** Representative view of Western blot assay of E-cadherin and Vimentin in tumor tissue (T) and paired peri-tumor tissue (N) from HCC patients with up-regulated Brachyury in tumor. **(D)** Scatter plot with fitted values intervals for T/NT (ratio of tumor tissue and paired peri-tumor tissue) expression of Bachyury and Vimentin. **(E)** Scatter plot with fitted values intervals for T/NT expression of Bachyury and E-cadherin.

As EMT has been accepted as a potential mechanism underlying cancer metastasis, we explored the role of Brachyury in regulating EMT in HCC cells, Up-regulation of Brachyury in HepG2 cells resulted in the decreased expression of epithelial markers (E-cadherin and γ-catenin) and increased expression of mesenchymal marker (snail), as evidenced by immunofluorescence staining (Figure 4A). Western blot analysis also revealed that the protein level of vimentin (mesenchymal marker) was increased, while E-cadherin and γ-catenin were decreased in Brachyury-overexpressing cells compared with control cells (Figure 4B).

A similar result was observed in tissue samples of HCC. The expression of E-cadherin and vimentin in 40 HCC samples were analyzed using Western blotting. As shown in Figure 4C, high expression of vimentin (T/NT ≥ 2) accompanied by low expression of E-cadherin (T/NT < 0.5) was detected in HCC specimens with Brachyury overexpression (T/NT ≥ 2). High expression of vimentin and low expression of E-cadherin was detected at 79.2% (19/24) and 66.7% (16/24) in high-Brachyury expression HCC specimens (T/NT ≥ 2), respectively. Conversely, high expression of vimentin and low expression of E-cadherin was only detected at 31.3% (5/16) and 25.0% (4/16) in low-Brachyury expression HCCs (Table 3). Moreover, by linear analysis, it was found that Brachyury expression was positively correlated with vimentin (p < 0.001) and inversely correlated with E-cadherin (p < 0.01) (Figure 4D,E). These data further support the involvement of Brachyury in EMT as observed in HCC cells.

**Table 3:** **Relationship between Brachyury expression and vimentin/E-cadherin in 40 HCC tissues**

		**Vimentin T/NT**		**E-cadherin T/NT**	
		**≥2**	**<2**	**>0.5**	**≤0.5**
Brachyury T/NT	≥2	19	5	8	16
	<2	5	11	12	4
p-Value			0.001		0.01

### Brachyury regulates EMT via PTEN/AKT/Snail pathway

**Figure 5:**
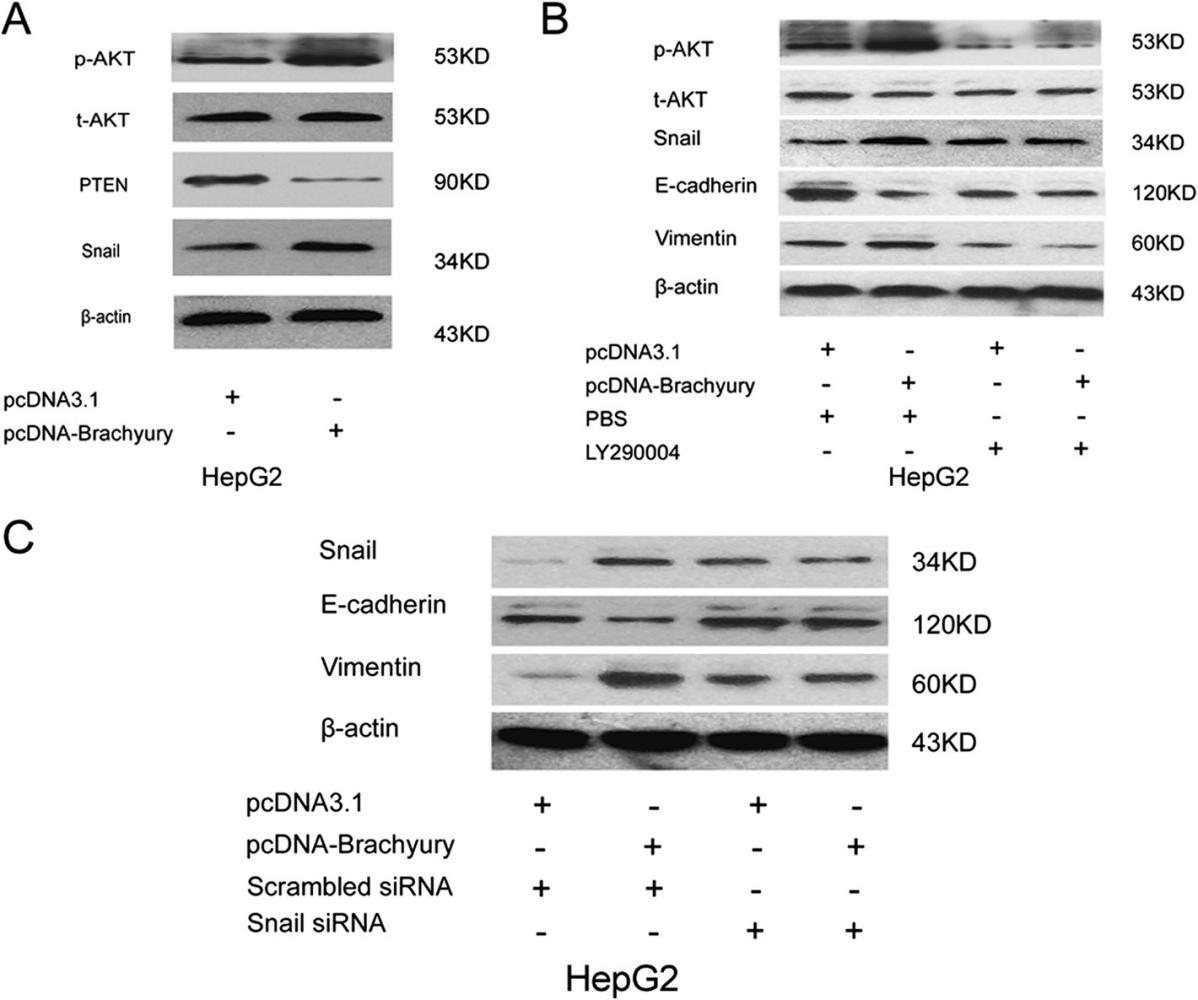
**Bachyury regulates EMT via Akt/Snail pathway. (A)** Western blot assay demonstrated overexpression Bachyury repressed PTEN and enhanced p-Akt, Snail, but not t-Akt expression in HepG2 cells. **(B)** AKT inhibitor prevented the Brachyury-mediated increase in p-AKT and Snail expression and abolished Bachyury-induced HCC EMT phenotype. **(C)** SiRNA against snail abolished Bachyury-induced Snail expression and HCC EMT phenotype.

Numerous studies have suggested that Akt activation plays a pivotal role in tumor progression via induction of EMT [26-29]. Thus, the Akt activation is usually up- and downregulated by tumor suppressor PTEN. Western blot revealed that PTEN expression was significantly influenced by Brachyury overexpression (Figure 5A), and it confirmed the involvement of PTEN in Brachyury-mediated Akt activation. Snail has been documented to be regulated by Akt and is a predominant mediator of EMT [30,31]. To test if Snail was involved in Brachyury-mediated EMT, the effect of Brachyury on Snail activation was examined. As expected, expression of Snail was significantly increased in cells with Brachyury-overexpression.

The Akt/Snail pathway involvement in EMT-mediated Brachyury-overexpressed HCC cells was investigated. Brachyury-HepG2 cells were treated with Akt inhibitor (LY290004) and small interfering RNA (siRNA) against Snail, and the effects on cell EMT were determined as described previously. As shown in Figure 5, treatment with Akt inhibitor prevented the Brachyury-mediated increase in p-Akt and Snail (Figure 5B). Transfection with siRNA against Snail effectively abolished Brachyury-induced Snail expression (Figure 5C). The expression of E-cadherin was correspondingly increased while vimentin was reduced in Brachyury-transfected cells after treatment with Akt inhibitor or Snail siRNA. These results indicate that Brachyury mediates the mesenchymal transition of HCC cells through the Akt/Snail pathway.

## Discussion

Recently, accumulating evidence has suggested that T-Brachyury plays an important role in carcinogenesis and metastasis of multiple types of human cancers, and may serve as a prognosis marker. Nerbil et al. found that early stages colorectal cancer patients (T1-2N0M0, Dukes A) showed a significantly decreased survival when Brachyury was expressed in the tumour tissue while no correlation was observed in later tumour stages, suggesting a possibility to detect metastatic cancers during early stages of colorectal carcinogenesis by using an antibody against brachyury in immunohistochemistry analysis [18]. Brachyury was shown to be overexpressed in human lung tumors [32]. Furthermore, Brachyury mRNA expression in primary lung carcinoma tissues was demonstrated as a significant predictor of poor prognosis for 5-year disease-free survival and overall survival rates and was significantly correlated to vascular invasion, lymphatic permeation, histological grade, pathologic T stage, and pathologic N stage [33]. In addition, Palena and colleagues also revealed that overexpression of brachyury in breast carcinomas is associated with poor prognosis [34]. More recently, Brachyury was shown to be overexpressed in prostate cancer and metastatic tumors when compared with normal tissues and play an important role in prostate cancer aggressiveness [35]. Here, we presented for the first time that the Brachyury was highly expressed in HCC tissues compared with adjacent nontumorous tissues; Brachyury expression was correlated with tumor size (P = 0.037), intrahepatic invasion (P = 0.025), distance metastasis (P = 0.039), and outcome of HCCs, adding to the evidence that Brachyury may serve as a prognosis marker and potential therapeutic target for HCC.

The EMT usually occurs in the critical phases of embryonic development and organ fibrosis. This program has also been shown to be the pivotal mechanism contributing to cancer metastasis [36]. It is well established that EMT gives rise to the dissemination of single carcinoma cell from the site of the primary tumor [37]. The EMT has also been reported to be involved in the progression of HCC and correlates with the prognosis of cancer metastasis [12]. Recently, Brachyury has been identified as an important EMT regulator by modulating Slug and E-cadherin expression in various tumor cells; however, whether Brachyury promotes hepatoma cells EMT and cancer metastasis is not clear so far. The study data suggest that Brachyury promotes hepatoma cells metastasis through modulation of its EMT phenotype. This notion was based on the findings as follows: (1) Mesenchymal markers were significantly upregulated in the Brachyury overexpression HCC cell lines, whereas the epithelial markers were remarkably decreased by immunocytochemical analysis. (2) These results were further confirmed by immunostaining analysis of cultured cells and HCC tissues. (3) In vitro and in vivo metastasis assays demonstrated that enforced Brachyury enhanced the metastasis potential of HCC cells and induced more metastatic lesions in liver of nude mice. Therefore, these experimental data indicated that Brachyury might promote HCC metastasis through, at least partially, induction of EMT in hepatoma cells.

Brachyury was recently demonstrated to induce EMT in human epithelial cells through repression of E-cadherin and induction Slug [20]. Another study demonstrates that Brachyury expression is enhanced during TGF-β1-induced EMT in various human cancer cell lines, and a positive feedback loop is established between Brachyury and TGF-β1 in mesenchymal-like tumor cells. Interestingly, Brachyury overexpression could promote TGF-β1 upregulation by activating its promoter, while inhibition of its signaling could decrease Brachyury expression, induce a mesenchymal-to-epithelial transition, and provide increased susceptibility to tumor cells towards chemotherapy [38]. The present study focused on the regulation of AKT pathway, because increasing evidence had demonstrated that activated AKT pathway could play a central role in EMT process and tumor metastasis [26-29]. Activation of Akt in Brachyury-induced EMT was observed in the study, correlating with vimentin induction and E-cadherin downregulation. Further, Snail has been documented to be regulated by Akt, and it has been a predominant mediator of EMT [30,31]. The experimental data of present study showed that expression of Snail was significantly increased in cells with Brachyury overexpression. Additionally, treatment with Akt inhibitor prevented the Brachyury-mediated increase in p-Akt and Snail, and transfection with siRNA against Snail effectively abolished Brachyury-induced Snail expression. These experimental data provide evidence that Brachyury could promote EMT of hepatoma cells via Akt/Snail-dependent pathway. It was hypothesized that Brachyury might activate cytoplasmic Akt signal pathway to repress the tumor suppressor PTEN. Overexpression of PTEN has been reported to strongly inhibit the EMT of mesoderm cells. The functions of PTEN as a negative regulator of the PI3K/Akt pathway via dephosphorylation of PtdIns(3,4,5)P(3) has been reported, participating in regulation of EMT during cancer progression [39]. The study data demonstrated that PTEN expression was significantly influenced by Brachyury overexpression. However, more evidence is required to confirm whether PTEN expression is directly regulated by Brachyury.

To summarize, Brachyury could play an important role in HCC metastasis by EMT induction via, at least partially, PTEN/AKT/Snail-dependent pathway; and it may serve as a valuable prognostic biomarker and potential therapeutic target. The present study results uncovered a novel function and molecular mechanism for Brachyury in HCC which will shed new light on the understanding of tumor progression and metastasis.
